# Cholesterol-dependent amyloid β production: space for multifarious interactions between amyloid precursor protein, secretases, and cholesterol

**DOI:** 10.1186/s13578-023-01127-y

**Published:** 2023-09-13

**Authors:** Vladimir Rudajev, Jiri Novotny

**Affiliations:** Department of Physiology, Faculty of Science, Charles University, Prague, Czech Republic

**Keywords:** Amyloid β, Amyloid precursor protein, Amyloidogenesis, Cholesterol, Secretase

## Abstract

Amyloid β is considered a key player in the development and progression of Alzheimer’s disease (AD). Many studies investigating the effect of statins on lowering cholesterol suggest that there may be a link between cholesterol levels and AD pathology. Since cholesterol is one of the most abundant lipid molecules, especially in brain tissue, it affects most membrane-related processes, including the formation of the most dangerous form of amyloid β, Aβ42. The entire Aβ production system, which includes the amyloid precursor protein (APP), β-secretase, and the complex of γ-secretase, is highly dependent on membrane cholesterol content. Moreover, cholesterol can affect amyloidogenesis in many ways. Cholesterol influences the stability and activity of secretases, but also dictates their partitioning into specific cellular compartments and cholesterol-enriched lipid rafts, where the amyloidogenic machinery is predominantly localized. The most complicated relationships have been found in the interaction between cholesterol and APP, where cholesterol affects not only APP localization but also the precise character of APP dimerization and APP processing by γ-secretase, which is important for the production of Aβ of different lengths*.* In this review, we describe the intricate web of interdependence between cellular cholesterol levels, cholesterol membrane distribution, and cholesterol-dependent production of Aβ, the major player in AD.

## Background

Numerous pathological processes have been described that may play a role in the etiology of Alzheimer’s disease (AD). Many proteins have been discovered that link AD pathology to cell membrane composition, transport, and dynamics, suggesting a critical role of membranes in brain health. These include phosphatidylinositol-binding clathrin assembly protein (PICALM), sortilin-related receptor 1 (SORL1), clusterin (ApoJ), bridging integrator 1 (BIN1)*,* ATP-binding cassette (ABC) transporters, and ApoE, which is mainly responsible for the intercellular transport of cholesterol in the brain, with the ApoE4 isoform being the most common element associated with the sporadic form of AD [1–6]. According to the amyloid hypothesis of AD, altered processing of the amyloid precursor protein (APP) to amyloid β (Aβ) peptides dictates the onset and progression of both familiar (FAD), and the much more prevalent sporadic form of AD [7–9]. Among the hallmarks of AD associated with aberrant Aβ formation and aggregation of the most dangerous 42-amino acid long Aβ42, increased reactive oxygen species (ROS) production, mitochondrial dysfunction, calcium dyshomeostasis, and apoptosis have been reported most frequently [8, 10–14]. In addition, endosomal-lysosomal dysregulation [15–21] and altered autophagy and mitophagy are also associated with increased Aβ production and Aβ42 action [22–27].

During the development of AD, the content of cholesterol, cholesterol esters, oxysterols, peroxidized lipids, or ceramides may increase or decrease depending on the brain part, specific membrane, or disease stage [6, 28–37]. Changes in lipid populations in the brain reflect alterations in lipid synthesis, lipid degradation, lipid clearance, lipid transport, and also the extent of blood–brain barrier (BBB) disruption associated with increased permeability to cholesterol-containing lipid particles. Disruption of BBB integrity caused by elevated plasma cholesterol leads to lipid dyshomeostasis in the brain and links total body cholesterol levels and its regulation to brain health [38–41].

Cholesterol plays multiple roles in Aβ-mediated AD pathology, and a high-cholesterol diet is associated with AD-related symptoms [39, 41–47]. However, several studies have found an inverse correlation or no association between cholesterol intake and Aβ production or dementia progression [48–51]. The inconsistency of results could be due to the use of different experimental designs and AD models, the nonlinear relationship between plasma and brain cholesterol levels, the magnitude of cholesterol changes and differences in monitoring cholesterol as total pool, LDL- or HDL-bound cholesterol, membrane cholesterol, or cholesterol in different parts of the brain. Age, sex, presence of vascular disease, or presence of the risk ApoE4 allele and other AD-related genes may also influence the effects of cholesterol on the disease [48, 50, 52, 53].

One of the most important approaches to studying the role of cholesterol in AD has been the use of statins, which inhibit a key enzyme in cholesterol synthesis, 3-hydroxy-3-methyl-glutaryl-coenzyme A reductase. Although many studies showed an ameliorative effect of statins on AD pathology [54–58], the pleiotropy of statin action, which also affects protein prenylation involved in vital cellular and physiological processes, may complicate interpretation of results [59, 60]. However, authors often use alternative methods to lower cholesterol levels, such as the use of methyl-β-cyclodextrin (MβCD), which emphasize the importance of cholesterol in AD. Cholesterol undoubtedly influences Aβ-membrane interactions and its possible toxic effects [61–67]. Cholesterol levels are also associated with Aβ degradation and clearance, as these mechanisms depend on cholesterol-rich lipid rafts and cholesterol-transporting proteins, including ApoE and ABC pumps [68, 69]. Finally, the presence and amount of cholesterol dictates the mode of APP processing and the resulting amyloid forms that determine the fate of cells and organisms. The present review focuses on the role of cholesterol and cholesterol-dependent membrane rafts in APP processing that determines the amount and forms of amyloid β in the brain.

## Cholesterol

### Cholesterol pools

Although the human brain represents only 2% of body weight, it contains about 25% of total body cholesterol. Since cholesterol cannot normally pass the BBB, its homeostasis in the brain is regulated mainly by glial cells. During postnatal development, massive cholesterol synthesis in the brain is connected with a brief period after birth when myelination by oligodendrocytes occurs [70, 71]. In adulthood, cholesterol turnover in the neuronal system is relatively slow because most cholesterol is bound in the myelin sheaths [40, 69, 72–74]. However, cholesterol turnover is not the same throughout brain tissue [75–77]. Therefore, small age- or disease-related changes in cholesterol content in neuronal membranes may appear invisible when all brain tissue is analyzed. While myelin-rich white matter has the slowest cholesterol turnover and glial cells the intermediate, active neurons are characterized by the highest cholesterol metabolism [75, 78]. Although essentially all brain cells are capable of producing cholesterol, the needs of mature neurons are met primarily by astrocytes [38, 77, 79–81]. Mature neurons have a limited ability to synthesize cholesterol and rely on a supply of the cholesterol-rich lipoprotein ApoE from astrocytes [79, 82]. Axons in particular lack the ability to synthesize cholesterol, although the soma shows limited cholesterol synthesis in culture [83, 84]. Because axons are often very long, transport of cholesterol from the soma may be insufficient to supply active synaptic membranes. Therefore, local synaptic demand is met by astrocytic ApoE particles released in close proximity to ApoE receptors on the neuronal plasma membrane (PM). This mechanism not only conserves neuronal energy but also provides rapid regulation: ApoE receptors are enriched in the neuronal PM during increased neuronal activity, and on the other hand, excess oxidized cholesterol produced by neurons can rapidly reduce cholesterol synthesis in astrocytes through feedback mechanisms [82, 85]. In remyelination processes, microglia, oligodendrocytes, and neurons play the main role in cholesterol production rather than astrocytes [71, 86]. These observations underscore the fact that cholesterol requirements are saturated by different cell populations locally and depending on the (patho)physiological conditions and ontogenetic stage [38, 70, 71, 76, 77, 80, 86].

There are several cholesterol pools in cells that can be altered in AD. Synthesis of cholesterol in the endoplasmic reticulum (ER) is associated with transport to the Golgi apparatus (GA) and PM [87]. Extracellular (e.c.) cholesterol is bound to cholesterol-rich lipoproteins. In the brain, the ApoE protein of astrocytic origin is the major platform for the organization of lipoproteins that enter the cell by endocytosis [82, 88]. Conversely, the accumulation of sterol molecules in the PM is associated with cholesterol and oxysterol efflux into the ApoE lipoprotein particles, which is assisted by ABC transporters [89–91]. Cholesterol can also be released from cholesterol esters concentrated in lipid droplets by the action of ER-resident acyl-CoA cholesterol acyltransferase (ACAT). During aging and neurodegeneration, lipid droplets accumulate in astrocytes and affect cholesterol homeostasis in the cell [89, 92]. Quantitatively, about 1% of cellular cholesterol is converted to esters, while 40% is oxidized. The rest is mainly localized in the PM or exported via the ABC-lipoprotein system [38]. In the PM, cholesterol is predominantly concentrated in lipid rafts. Lipid rafts have been described as PM regions rich in cholesterol and sphingolipids (SL), especially sphingomyelin (SM), and characterized by a specific protein composition [93–98]. A higher proportion of saturated and long carbon chains and planar cholesterol molecules results in a less fluid and more rigid liquid-ordered (Lo) phase, which, together with a higher raft thickness, strongly affects the nature of membrane interactions with both peripheral and transmembrane (TM) proteins [99].

### Distribution of cholesterol in membrane bilayers

There are seemingly conflicting data and views on the preference of cholesterol for the cytofacial or exofacial leaflet of the membrane [100]. Experimental and theoretical considerations led to the conclusion that cholesterol is enriched in the less fluid outer layer where it interacts with SM [101–107]. At lower concentrations (15%), most of the cholesterol is captured by SM molecules in the outer leaflet. However, cholesterol content of 33% is sufficient to saturate the SM binding sites, and the cholesterol is evenly distributed [108]. This means that cholesterol distribution is affected by other membrane lipids, which may be different in different tissues, age groups or stages of the disease. Other authors observed preferential accumulation of cholesterol in the cytofacial leaflet [109–112]. Dynamic redistribution from the exofacial [113] or the cytofacial [114] leaflet may occur after statin or ethanol administration and as a result of changes in polyunsaturated fatty acids (PUFA), aging, or changes in the function of cholesterol transport proteins [109, 113–115].

Cholesterol may have a stabilizing effect on the inner membrane leaflet by reducing the bending energy of the inverted hexagonal phase, which consists mainly of cytofacial phosphatidylethanolamine (PE) [116]. The dependence of cholesterol distribution on the length of the hydrocarbon chain of SM was observed in model systems and in erythrocytes. Only the long chain C24 SM, but not the shorter chain C16 SM interdigitated from the outer to the inner monolayer, where it formed hydrophobic holes filled with cholesterol molecules and caused a redistribution of cholesterol from the outer to the inner monolayer [117]. Similar results on the distribution of sterols in the bilayer were obtained in yeast [118]. Thus, the nonuniform localization of SL in exofacial raft patches also leads to cytofacial aggregation of cholesterol in the raft fraction but not in the raft surroundings. However, the rapid cholesterol flip/flop movements and the use of different experimental models make it difficult to draw a definitive conclusion [106, 107]. Because particular membranes in cells, tissues, and organs vary considerably in composition, cholesterol molecules may respond dynamically to the presence of other lipid populations with particularly long saturated or unsaturated fatty chains [107–109, 116–119]. This uncertainty complicates the interpretation of cholesterol effects on Aβ synthesis, and furthermore, only long-term equilibria in cholesterol distribution need to be considered because AD is a long-term pathology.

### Oxysterols

In addition to cholesterol, oxysterols (oxidation products of cholesterol) are also involved in the intertwining of Aβ production and cholesterol metabolism [36, 120]. Excess cholesterol in the brain is oxidized to 24-hydroxycholesterol (24-OHC), which, unlike cholesterol itself, exits the brain via the BBB, a process that is in equilibrium with cholesterol synthesis in the brain [75, 121–123]. 24-OHC has been shown to be elevated in the brain of AD patients along with plasma-derived 27-hydroxycholesterol (27-OHC), with 24-OHC levels decreasing in the later stages of the disease [30, 88, 124, 125]. Elevated brain oxysterols may trigger neuronal excitotoxicity and neuroinflammation and serve as one of the markers for AD [126, 127]. Cholesterol oxidation leads to membrane thinning and a decrease in membrane order [128], which is accompanied by increased Aβ-membrane association [129]. The flux of 27-OHC from plasma to brain forms is one of the links between AD and hypercholesterolemia [124, 130], while a reduction 27-OHC in serum alleviates symptoms of the disease [131].

However, the role of 24-OHC in brain health remains controversial, as its effects are highly dependent on oxysterol concentration and experimental model [30, 132]. Both oxysterols, but especially 24-OHC, were found to inhibit Aβ synthesis in neurons [133]. It is worth noting that 24-OHC may act as an activator of liver X receptors that induce transcription of cholesterol export pathway genes (ABCA1, ABCG1, ApoE). Importantly, enhanced cholesterol oxidation was associated with decreased cholesterol content and cholesterol-dependent Aβ production [38, 134].

### ApoE

ApoE plays a crucial role in cholesterol delivery from astrocytes to neurons and has been found to be involved in AD pathology at many levels in complex ways [135–138]. Of the three ApoE variants, ApoE4, which contains Arg112/Arg158 instead of Cys112/Cys158 (ApoE2) or Cys112/Arg158 (ApoE3), is associated with AD [139–146]. ApoE4 impairs lipid transport between cells, reduces Aβ clearance, and promotes Aβ aggregation into toxic conformations [136, 142, 147–149]. ApoE4 is thought to be less lipidated than ApoE2 and ApoE3 [121, 149, 150]. Thus, ApoE4 does not sufficiently remove cholesterol from the exofacial leaflet of the PM. Accordingly, ApoE4 knock-in mice exhibiting cholesterol-induced amyloid pathology showed a ∼ 2-fold increase in cholesterol in the exofacial leaflet compared with wild-type or ApoE3 mice [141]. In human ApoE4-expressing astrocytes derived from induced pluripotent stem cells, accumulation of cholesterol, and higher production but less efficient clearance of Aβ were observed [147]. Moreover, ApoE4 enhanced endosomal dysfunction by decreasing the amount of the Na^+^/H^+^ exchanger NHE6 and lowering pH in endosomes where ApoE-bound Aβ and its receptor LRP1 remain trapped, resulting in decreased amyloid clearance [151–153].

### ABC transporters

ATP-binding cassette (ABC) transporters are responsible for the export of molecules from cells, and their dysregulation leads to degenerative brain disorders [154, 155]*.* Among the ABC transporters, ABCA1 and, in a lesser extent, ABCG1 and ABCG4 are particularly required for cholesterol efflux because they are responsible for lipidation of ApoE [38, 88, 155–157]. Whereas ABCA1 and ABCG1 are localized in astrocytes, ABCG4 is preferentially expressed in neurons, and ABCA7 in microglia [121, 154, 158]. The increase in ABCA1 content in the PM is accompanied by increased cholesterol export and more lipidated apoE particles, and vice versa [149, 159]. However, in cultured astrocytes and neurons, Aβ42 induced an increase in cholesterol synthesis and ABCA1 content but did not result in increased production of lipidated ApoE [160]. Because the function of ABC transporters, ApoE lipidation, cholesterol, and Aβ transport capacity are interconnected, disruption of cholesterol regulation at any level may lead to severe brain dysfunction [154, 161].

### Cholesterol changes in AD

The lipid composition of brain tissue has been found to change during aging or AD progression. Age-dependent enrichment of the neuronal PM membrane with cholesterol and SM-dependent lipid rafts was associated with higher Aβ-induced cell injury [162, 163]. Cholesterol content increased in the PM of brain areas characterized by AD-associated damage during aging [29, 35, 164]. Elevated cholesterol and ganglioside GM1 levels were observed in synaptosomes isolated from cortices of AD patients [31], which was associated with increased Aβ generation [165]. In combination with the ApoE4 genotype, increased cholesterol content in the frontal cortex was closely associated with senile plaque formation [28, 166]. The accumulation of cholesterol in mitochondria makes mitochondrial membranes less permeable to cytosolic glutathione, which is associated with increased Aβ-induced oxidative stress [167, 168]. On the other hand, some studies show opposite results. A 30–35% reduction in cholesterol content was observed in the temporal gyrus or hippocampus of AD patients [169, 170], which was associated with a thinning of the bilayer by 0.4 nm [170]. The AD-associated demyelination was also associated with a ~ 12% loss of cholesterol [73]. At this point, it must be emphasized that not only changes in total cholesterol content but also specific changes in different membrane compartments or raft fractions may influence the progression of AD. In addition, cholesterol has many effects on the membrane that influence: (1) membrane thickness and fluidity, (2) interactions of enzymes with substrates and activation of receptors, (3) enzyme activities, and (4) protein sorting and internalization [44, 165, 171, 172]. With respect to AD and as described below, the processing of APP and the formation of Aβ can also be directed by cholesterol content and distribution in the cell [74].

## Amyloid β formation

### Amyloid β peptide

Aβ is a 37–43 amino acid long peptide, with Aβ40 representing the absolute majority of amyloid products and the ten times rarer Aβ42 known to be the most dangerous form in the brain. All Aβ peptides originate from the amyloid precursor protein, a type I single-span TM protein, which is specifically cleaved by α-, β-, and γ-secretases at distinct sites [173–179]. Aβ is one of the intrinsically unstable polymers that can adopt different spatial arrangements with varying degrees of toxicity [180, 181]. Thus, Aβ peptides can self-organize into oligomers capable of disrupting the membrane or initiating destructive processes in cells [8, 17, 18, 182–187]. The membrane environment, with specific lipids and proteins, co-establishes the extent of amyloid toxicity by creating conditions under which Aβ aggregates into cell-damaging elements. Among lipids, gangliosides GM1, SM, and cholesterol form binding platforms for Aβ [31, 66, 67, 188–191].

### Amyloid precursor protein

**Fig. 1:**
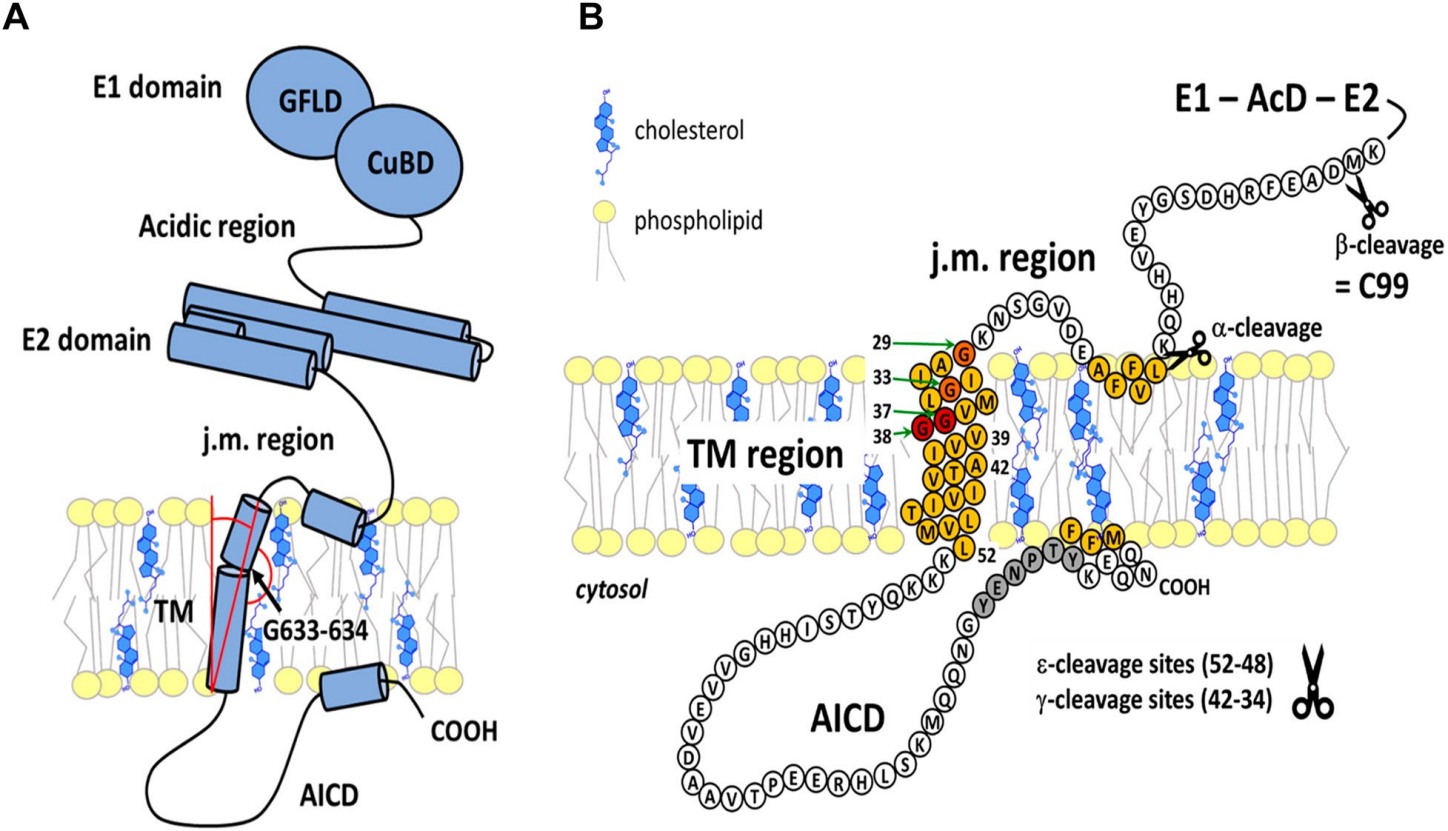
Amyloid precursor protein. **A** The extracellular (e.c.) part of APP consists of the E1 domain [containing the N-terminal growth factor-like domain (GFLD) and the copper-binding domain (CuBD)], the acidic region, and the E2 domain. The juxtamembrane (j.m.) region connects the e.c. portion to the transmembrane (TM) segment. The C-terminus is cytosolically aligned at the end of the cytosolic APP intracellular domain (AICD). In the TM domain, glycines G633-634 form a kink that is locally destabilizes the secondary structure. The red lines show the angles characteristic of the orientation of APP in the membrane (see text). **B** Amino acid sequence of the TM segment and AICD of APP. Cleavage sites for α-, β-, and γ- secretase (including the ε-site) are indicated. Light orange—amino acids buried in the hydrophobic zone of the bilayer, dark orange and red—glycines forming glycine zippers (dimerization motifs). Glycines 37/38 of C99 (after β-cleavage) correspond to G633/634 of APP (**A**). Adjusted according to [193, 195, 201, 203, 256, 338]

The role of APP in nervous system development and function is underpinned by its ability to bind the e.c. matrix and mediate intercellular contacts [192–194]. Of the three known isoforms, APP695, which contains 695 amino acids, is most abundant in the brain, where it serves as a precursor for Aβ synthesis. The structure of APP is characterized by a large e.c. glycosylated N-terminus, a single TM α-helix, and a short C-terminal portion [175, 192]. The e.c. part of APP695 consists of the E1 domain (Leu18-Ala190) and the E2 domain (Ser295-Asp500) connected by the acidic domain (AcD) (Fig. 1). The α- and β-secretase cleavage sites are located in the juxtamembrane (j.m.) region connecting the E2 domain to the single TM helix. Both the E1 and E2 domains contain a heparin-binding site that allows the e.c. portion of the molecule to participate in interactions with the e.c. matrix and also in trans interactions with APPs on neighbouring cells [195–199]*.* A short α-helix, representing an extrinsic anchor of APP, may form at the j.m. region, which is buried in the membrane surface by the hydrophobic sequence Val-Phe-Ala [200–202]. Similarly, the C-terminus (the cytosolic APP intracellular domain—AICD) may also be dynamically anchored in the bilayer as an amphipathic helix, which may affect the accessibility of the YENPTY motif required for endosomal sorting or AICD release [200, 201, 203]. The TM domain of APP contains 24–26 amino acids (Asn623/Gly625-Leu648/Lys649) arranged predominantly in a right-handed α-helix depending on the environment [193, 200, 204–208]. Gly633–Gly634 are responsible for the relatively high flexibility of this region, which is required for APP processing, TM domain dimerization, and interactions with cholesterol [204, 206, 209]. Two angles characterize the position of APP in the bilayer. The first describes the overall TM α-helix tilt and the second the hinge angle caused by the glycine Gly633–Gly634 (Fig. 1A). Both angles are affected by the membrane thickness, which in turn affects the flexibility of APP, the stability of the secondary structure, and the accessibility of the cleavage sites for γ-secretase [203, 210, 211].

### APP processing by secretases

The APP molecule can be cleaved by three proteases, termed α-, β-, and γ-secretases. The α-secretase cleaves APP at the PM extracellularly releasing soluble sAPPα and the C-terminal fragment (CTF) C83. This enzyme belongs to the ADAM (a disintegrin and metalloproteinase) family, in which ADAM10 plays a dominant role in APP cleavage [212, 213]. However, only sequential proteolytic cleavage of APP by β- and γ-secretases leads to the formation of potentially deleterious Αβ peptide fragments. Intramembrane γ-secretase processing of C83 generates the nontoxic P3 peptide and cytosolic AICD with regulatory functions in the cell. The amyloidogenic process begins with APP cleavage by β-secretase, which generates soluble sAPPβ and the membrane-anchored CTF C99. In addition to AICD, sequential γ-secretase activity releases Aβ peptides of varying lengths depending on the position and orientation of the enzyme and substrate [175, 214–216].

### β-Secretase

The β-secretase is known as β-site APP cleaving enzyme-1 (BACE1), an aspartic protease whose enzymatic activity is highest in the low pH environment of intracellular (i.c.) organelles including the GA and endosomes, where amyloidogenic cleavage predominantly occurs [217–225]. Together with other members of the amyloidogenic cascade, part of BACE1 is concentrated in cholesterol-dependent lipid rafts [223, 226], and its enzyme activity is enhanced by cerebrosides, anionic lipids, and especially cholesterol [223]. Furthermore, artificial elevation of BACE1 in rafts led to increased Aβ production, which was reduced after cholesterol depletion, indicating cholesterol regulatory function [54]. The β-secretase cleavage of APP was enhanced after endocytosis of APP from the PM, resulting in coalescence of APP and BACE1-containing rafts in endosomes [227]. Besides endosomes, the GA is also associated with Aβ production. In AD, disruption of GA structure was associated with altered BACE1-APP accumulation in the GA as normal sorting mechanisms failed [228].

### γ-Secretase

The complex of γ-secretase consists of four subunits: presenilin (PSEN1/2), anterior pharynx defective (APH-1a/b), nicastrin, and the presenilin enhancer (PEN-2) [174, 221, 229, 230]. Whereas PSEN1 is widely distributed in cellular membranes, including the PM, PSEN2 is preferentially localized in the endosomal network and in the GA, where γ-secretase is localized in lipid rafts [230, 231]. Amyloidogenic processing of C99 by γ-secretase is concentrated in acidic organelles (mainly endosomes), where Aβ peptides can accumulate and form neurotoxic aggregates [8, 177, 218, 232–234]. Nicastrin sterically restricts the binding of APP to γ-secretase, so that only after cleavage of the e.c. APP moiety by α- or β-secretase does the active site of the presenilin complex gain access to the truncated substrate by recognizing the j.m. region of C99 [214, 235]. The γ-secretase cleaves the C99 stepwise, starting at the ε-site within the TM domain near the cytoplasmic face of the bilayer (Fig. 1B). Local destabilization of the TM α-helix is necessary for cleavage because the backbone carbonyl carbons must be exposed for nucleophilic attack by water molecules in the active site of the γ-secretase [236, 237]. Interaction of PSEN1 with the TM domain of C99 facilitates unwinding of the α-helix of C99 and transition to the β-sheet, which is the cleavable substrate for the secretase. Depending on the orientation of C99 in the bilayer, three to five cleavages usually follow, leaving mainly 3 but also 4–5 amino acid peptides. If the first ε-cleavage occurs at Aβ48 or 51, the final product is usually Aβ42, while ε-cleavage at Aβ49, 50, or 52 leads to Aβ40, although shorter variants can also be produced [178, 179, 214, 216, 236–240]. While the γ-secretase remains anchored in a stable position, the substrate (C99) shows flexibility that allows sequential peptide cleavage. Lys28 of C99 at the e.c. (or intraluminal) j.m. face blocks the shift of this peptide deeper into the membrane. When mutated to Ala, greater hydrophobicity allows movement of C99 closer to the active secretase and shorter amyloid fragments (Aβ34, 37, 38) are produced [241].

## Amyloid precursor protein

### APP trafficking and distribution

After synthesis, APP is diverted to the PM, where it undergoes nonamyloidogenic processing by α-secretase. Intracellularly localized Aβ production follows the internalization of APP from the PM [242, 243]. From endosomes, a pool of APP is transported back to the GA with help of SORL1. Depletion of SORL1 blocks transport of APP to the GA and keeps APP in endosomes, where it contributes to endosomal dysfunction and amyloidogenic APP processing instead of PM-localized α-secretase APP cleavage [4, 5, 244]. When APP is mutated at the sorting signal, it remains in the GA, resulting in reduced C99 production in endosomes that do not have a sufficient amount of APP. However, after a prolonged period of time, APP is also processed into C99 in the GA. leading to C99 i.c. accumulation because C99 is not sufficiently degraded in the endosomal-lysosomal compartment [245]. APP trafficking may also be affected by the extent of APP dimerization, as mentioned in the following section.

### APP dimerization

**Fig. 2:**
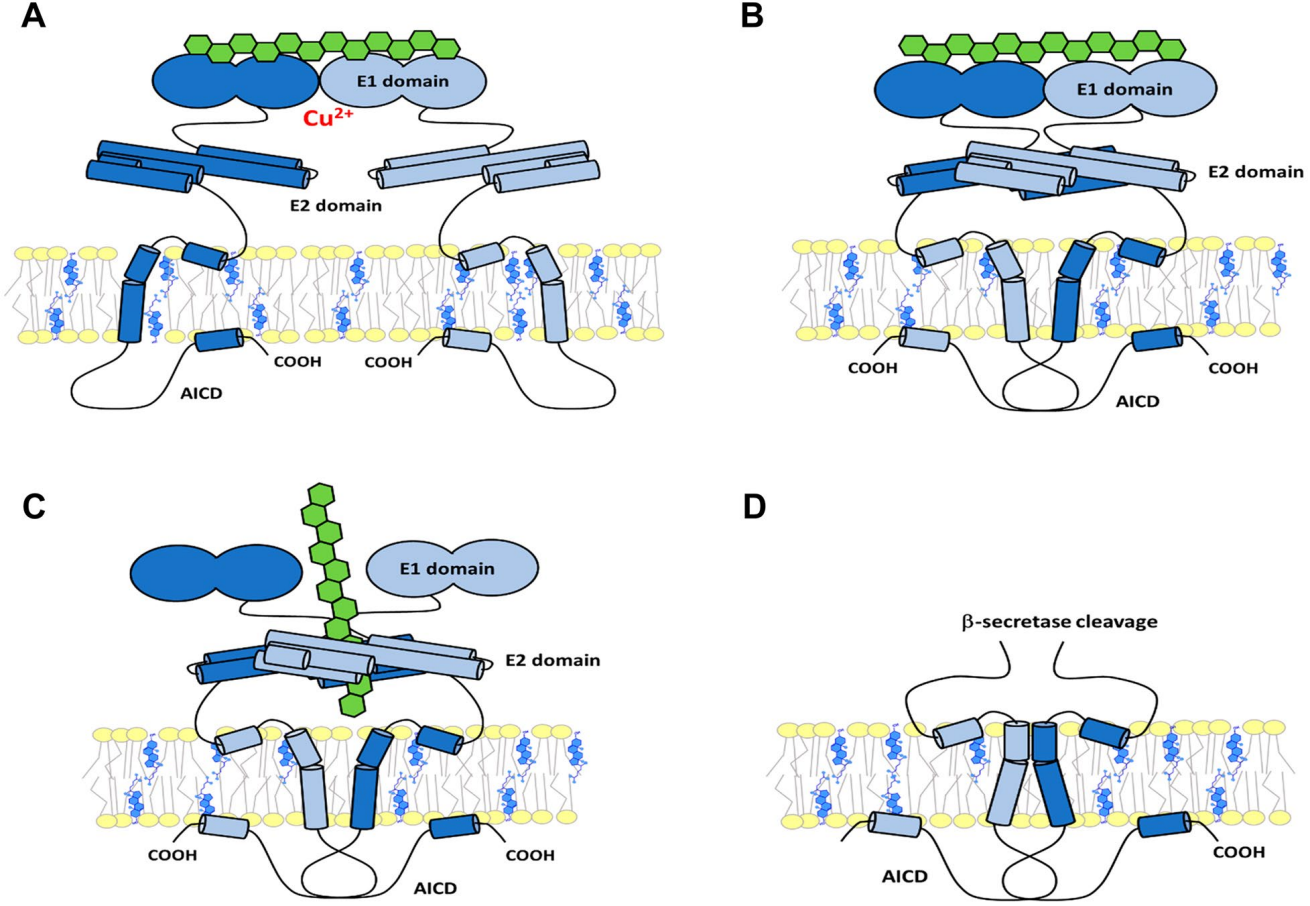
APP dimerization. **A** Dimerization via the E1 domain, which can be supported by heparan sulphate (green) or Cu^2+^ binding. **B** Distinct quaternary structure of APP dimer formed by heparan sulphate binding. **C** Dimerization of APP, triggered by heparan sulphate interaction with the E2 domain. **D** After shedding the e.c. part of the molecule by β-secretase, the TM domains approach each other and can dimerize. Adjusted according to [196, 198, 199, 250, 252, 255, 256]

APP contains several dimerization motifs, including the E1, E2, and TM domains (Fig. 2) [246–251]. Several models for the process of dimerization have been proposed. In any case, the e.c. domains appear to initiate the process and the TM domains can only approach after e.c. domain shedding by β-secretase [250, 252, 253]. Mutations in the TM dimerization glycine motifs do not affect α-secretase processing of APP [212]. The nature of dimerization and spatial orientation of full-length APP differs from cleaved C99, which lacks the e.c. dimerization domains that affect cellular localization and secretase processing. The dimeric status of APP may also be related to the APP-C99 environment, highlighting the importance of the site of Aβ formation. Consequently, the precise shape and position of APP-C99 in the membrane affects the production and length of the amyloid product [198, 247].

Because dimerization through the E1 and E2 domains is supported by e.c*.* heparan sulfate proteoglycans*,* the majority of PM-localized APP is in a dimeric form [196, 198, 199, 252, 254, 255]. Heparin-induced e.c. surface APP dimerization at neutral pH contrasts with i.c. accumulation of monomers, resulting from amyloidogenic processing predominant in the acidic endosomal environment [196, 199, 256]. Thus, the full-length APP forms more dimers than the cleaved CTF and the dimeric form is a preferred substrate for PM-located α-secretase [257]. Moreover, removal of the i.c. portion of APP reduces its internalization, and supports its dimerization and non-amyloidogenic processing at the PM [258]. However, dimerization of APP may also be associated with decreased α-secretase processing and increased amyloid production [259–262]. N-cadherin induced dimerization of the e.c. portion of APP and enhanced β-secretase-mediated cleavage of APP [259]. Dimerization induced by the binding of Cu^2+^ ions to the E1/E2 domain promoted Aβ generation, but with a lower Aβ42/40 ratio [263]. In contrast to monomers, the APP dimers showed reduced interaction with the sorting proteins SORL1 and LRP1, which interfered with the recycling of APP from the amyloidogenic environment of endosomes to the GA [244]. Increased SORL1 expression resulted in decreased Aβ production, as a greater amount of monomeric APP was transported to the GA and a smaller amount remained in the endosomal fraction in dimeric form as a preferred substrate for amyloidogenesis [264, 265]. At the same time, decreased amount of SORL1 led to accumulation of APP in endosomes and enhancement of the amyloidogenic pathway [264]. In another study, the concentration of APP in the trans-Golgi network (TGN) rather than in endosomes was associated with increased amyloid formation [266]. The authors suggested that APP must pass through the PM and be endocytosed during Aβ generation because most of BACE1 is concentrated in the endosomal compartment. After β-cleavage, C99 is delivered to γ-secretase and its processing occurs mainly in the TGN [266]. Because of the ambiguity of the data, multivalent effects of mutations must be considered, which are often used as a dimerization-modifying factor. In addition, it is necessary to assess the differences between APP and C99 dimerization. Dimerization of TM segments of C99 increases γ-secretase activity and Aβ formation but has no effect on BACE1-mediated APP processing [253]*.* Because of the different modes of APP-C99 dimerization, the resulting conformations may differ in their interactions with α, β, and γ-secretases [197]. It must be emphasized that both the monomeric and dimeric forms of C99 are substrates for γ-secretase processing, with the initial position for ε-cleavage and the arrest site for the enzyme depending strongly on the dimerization status and TM segment orientation in the bilayer [267]. Finally, not only the membrane position of APP-C99 plays a role in amyloidogenesis, but also its dimeric status and its occurrence in a specific environment enriched in certain secretases, which is a clear indication of the complexity of the process.

### C99 dimerization

**Fig. 3:**
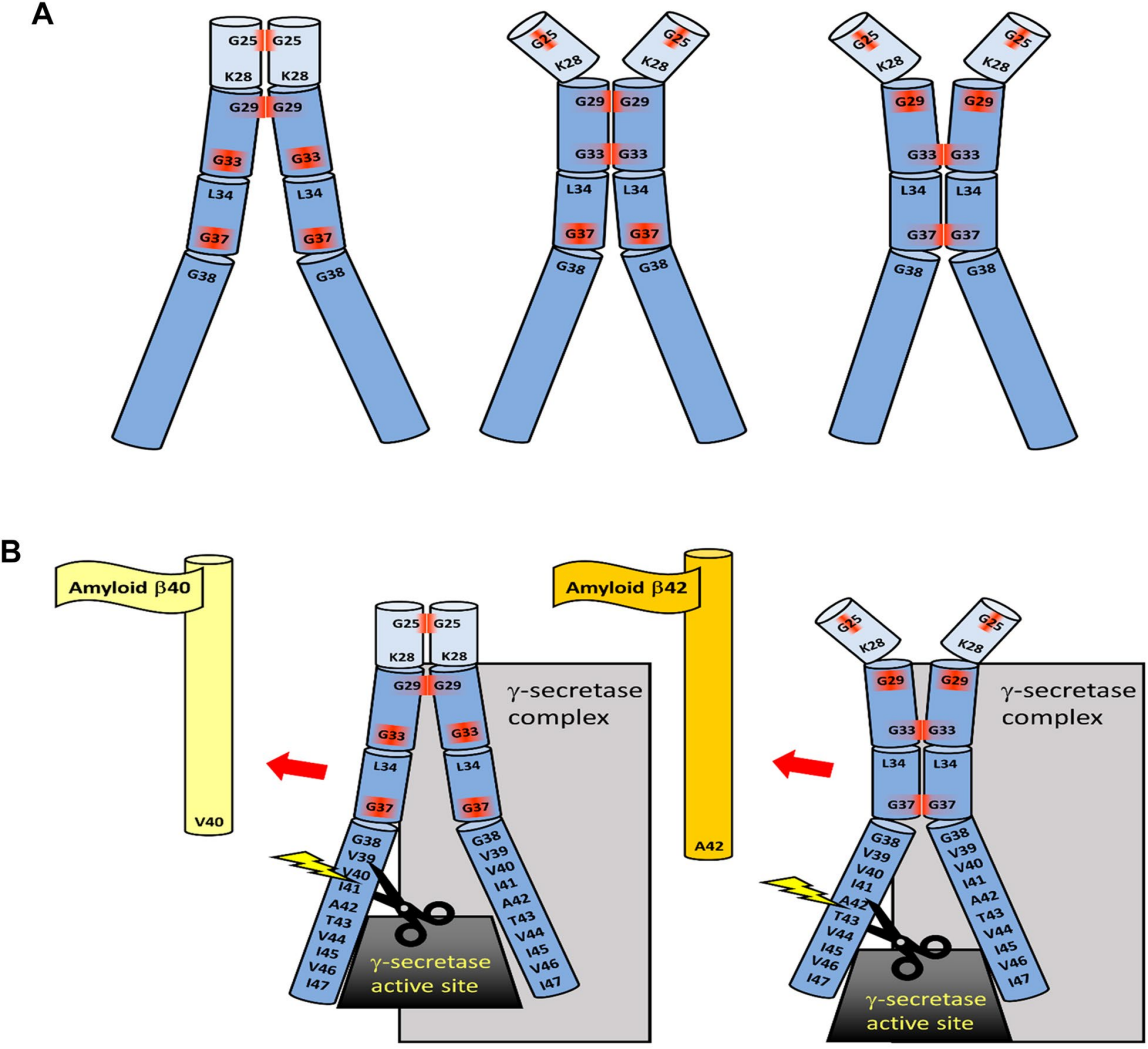
Dimerization of C99 and implications for γ-secretase-mediated processing. **A** Three glycine zippers are located in the TM segment and j.m. region of C99 (G25-x-x-x-G29; G29-x-x-x-G33; G33-x-x-x-G37/G38). Close positioning of the two monomers is required for dimerization. **B** Shifts and rotations of the TM helices caused by distinct Gly zipper interactions affect (1) the start cleavage site for γ-secretase because different amino acid sequences are exposed to the catalytic site, and (2) the stop of cleavage due to steric hindrance by tightly bound parts of the monomers. If the secretase cannot continue cleavage, longer and more dangerous amyloid β forms (amyloid β42) will be released. Adjusted according to [205, 237, 253, 267–270]

After APP cleavage by BACE1, the resulting C99 peptide can dimerize through three dimerization-inducing Gly-x-x-x-Gly motifs within the TM region (Fig. 3A) [237, 253, 267]. Computational models showed that the Gly33-x-x-x-Gly37/38 motif plays a major role in stabilizing the C99 dimer [205, 253, 268, 269]. However, Munter et al. emphasized the importance of the Gly29-x-x-x-Gly33 sequence, in which Gly33 is a crucial amino acid [267]. Each of the possible positions of C99 monomers in the dimer provides a distinct orientation to the γ-secretase, and diverse cleavage products are released as different starting ε-sites are recognized. The preferential action of PSEN1 on C99 dimerized through the j.m. Gly25-Gly29 motif leads to an Aβ40 product, whereas the TM dimerization through the Gly33-Gly37 sequence enhances i.c. PSEN2-mediated catalysis through the Aβ48-45-42 pathway [270] (Fig. 3B). TM glycines are known to disrupt the α-helicity and increase local peptide flexibility. In C99, in particular the Gly37-Gly38 pair forms a kink (Fig. 1) that increases the variability of the spatial arrangement of APP-C99 peptides in monomeric or dimeric structures, all of which can affect γ-secretase-mediated peptide processing [236, 247, 269, 271, 272].

**Fig. 4:**
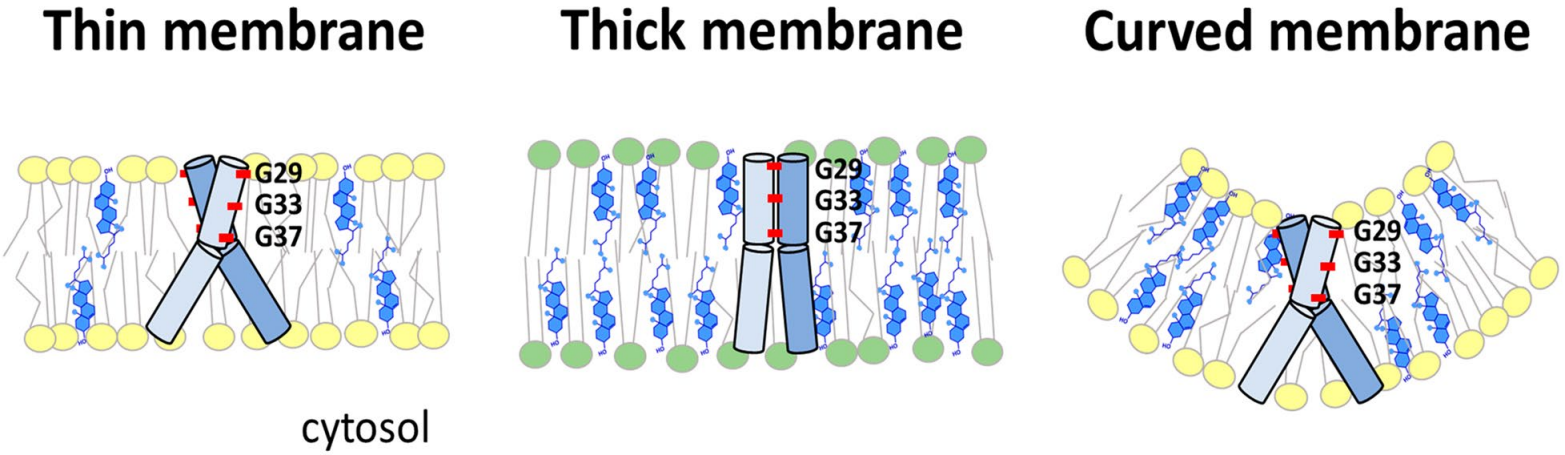
Membrane thickness and curvature affect the position of APP in the membrane. In thin or curved membranes, the C99/APP TM helices are more tilted than in thicker bilayers, which is associated with different types of dimerization and different γ-secretase-mediated processing. Adjusted according to [205, 206]

The dimers of C99 differ not only in the tilt position but, because the monomers can rotate, also in the surface area of the TM segments exposed to γ-secretase. Hence, if the lipid environment, membrane curvature, and bilayer thickness influence APP-C99 dimerization and orientation, they also have a strong impact on the production of Aβ species. In thinner membranes, the C99 dimer has a more open structure with more exposed glycine residues, whereas thicker membranes stabilize the “Gly-in” arrangements; in the thicker membrane, the TM regions are more parallel and the glycine sequences involved in dimerization are closer together than in the thinner membrane [205] (Fig. 4). Thus, membrane thickness, which is inherently different for raft and non-raft regions, as well as for the ER, GA, and PM, directly affects the processing of APP by influencing the association of APP and γ-secretase, γ-secretase activity, the start and end sites of the cleavage, and finally the representation of Aβ species [205, 206, 211]. Mutational studies showed that disruption of glycine zippers did not affect the interaction of APP with secretases, because AICD production remained undisturbed, whereas Aβ formation was reduced. Thus, ε-cleavage is not affected, but the altered orientation of the C99 peptide diminishes the γ-secretase-mediated cleavage system [253]*.* Due to the steric hindrance, γ-secretase ceases its activity toward C99 dimers earlier and a more dangerous Aβ42 is released, while C99 monomers are more easily cleaved to shorter products (Aβ38), as was shown by disruption of the TM glycine zippers [238, 248, 249, 267]. Apparently, any factor affecting Gly29-x-x-x-Gly33 dimerization, including lipid composition, elevated cholesterol, oxidative stress, or AD-associated mutations in PSEN1 and APP, may influence the site at which γ-secretase leaves the substrate and the variability of Aβ populations [267]. However, mutational studies involving glycine zippers should be taken with caution, as altered amino acid sequence may have a stronger effect on γ-cleavage than dimerization itself, leading to controversial results [215, 238, 249, 271, 273]. In several experiments, monomeric C99 was preferentially processed into longer Aβ42 (increased Aβ42/Aβ40 ratio), while the dimeric form gave rise to less dangerous shorter amyloid forms [251, 274]. Also, instead of glycine zippers, the 43TVIV46 motif in a TM region of C99 was found to be responsible for C99 dimerization [251]. Artificial C99 dimerization via the i.c. C-terminal domains decreased γ-secretase-mediated cleavage and Aβ production [275]. All these observations highlight the importance of the precise monomer orientation and mode of APP-C99 dimerization, as both the e.c. and TM domains of APP play distinct roles in amyloidogenesis.

## Cholesterol-dependent Aβ formation

### Lipid rafts as an environment for APP processing

Cholesterol- and SL-rich lipid rafts provide a platform that forms an optimal environment for APP-C99 interaction with β- and γ-secretases and for Aβ production [130, 223, 226, 276]. Only a small but physiologically relevant portion of APP is located in lipid rafts, where the amyloidogenic apparatus of β- and γ-secretase is predominantly localized [72, 277]. Normally, only 10% of APP is palmitoylated and processed by BACE1 in lipid rafts. Artificially increased or decreased APP palmitoylation results in elevated or reduced Aβ production, respectively [278].

All age- or disease-related changes in SM, cholesterol, or unsaturated fatty acids are associated with changes in raft composition and processes related to Aβ formation or Aβ degradation. In aged or AD brains, lipid alterations (especially the decrease in unsaturated fatty acids but also the increase in cholesterol) led to increased raft number and rigidity, accompanied by APP enrichment and enhanced APP-BACE1 association [184, 279–285].

### The relationship between Aβ production and cholesterol

Elevated cholesterol levels along with membrane-associated oxidative stress have been observed in the brain during AD; here, statins may alleviate symptoms of the disease [29, 34, 56, 58, 74, 286, 287]. Simulated cholesterol elevation in the PM of cultured neurons, cell lines, or mouse models caused increased association of APP-C99 with lipid rafts*,* enlarged endosomal compartment, impaired Aβ degradation, increased APP endocytosis, and Aβ42 secretion [143, 147, 167, 288–291]. Similarly, inhibition of cholesterol oxidase Cyp46a1 in mouse hippocampus resulted in increased cholesterol in neurons, increased APP recruitment to lipid rafts, Aβ formation, tau phosphorylation, endosomal enlargement, cognitive deficits, and hippocampal atrophy [292]. A shift from Aβ38 to Aβ42 generation was observed after increasing membrane cholesterol, indicating that γ-secretase leaves the substrate earlier [291]. In neurons, 70% cholesterol depletion did not affect α-secretase activity, but Aβ production was diminished, probably as a result of disruption of the APP-C99–β-/γ-secretase interaction [57, 293]. In cell cultures and primary hippocampal neurons, elevated cholesterol promoted APP raft localization, APP endocytosis, and Aβ42 secretion, which was reversible by cholesterol depletion [294, 295]. The APP internalization likely involves both clathrin- and caveolin/flotillin-dependent pathways, with cholesterol always increasing Aβ generation, indicating deleterious effects of disrupted cholesterol homeostasis in the brain [242, 243, 288, 294]. In addition to cholesterol-enhanced Aβ production, C99 accumulation may also represent a burdensome element in cellular pathology, adding to the complexity of cholesterol-mediated effects in AD [296].

### The effect of cholesterol on secretases

By forming lipid rafts, cholesterol creates optimal conditions for β- and γ-secretase activity [54, 165, 174, 201, 210, 223, 227, 297–299]. Cholesterol makes the active site γ-secretase more compact and supports secretase activity toward C99, which promotes Aβ42 generation [239, 300]. All four parts of the γ-secretase complex contain common TM cholesterol binding motifs CARC or CRAC [239, 301]. Blocking i.c. cholesterol transport impaired both β- and γ-secretase activities in SH-SY5Y cells and primary cortical neurons, where γ-secretase is concentrated in cholesterol-rich endosomal vesicles [230, 231]. Under nonpathological conditions, α-secretase cleavage predominates and is localized in the fluid cholesterol-poor membrane [201, 302, 303]. Moreover, α-secretase activity is inhibited by cholesterol [304]. Furthermore, a reduction of cholesterol content below 60% led to decreased internalization of APP, which was followed by increased association of ADAM10 and APP and nonamyloidogenic cleavage of APP [303]. The sex-specific effect of statins was observed in the model AD Tg2576 mice. Whereas females increased Aβ production by enhancing BACE1 activity, cholesterol lowering had no effect on APP processing in males. This suggests complex relationships between cholesterol levels, statin action (which also affects protein prenylation), and other factors in affected tissues [305]. Increasing dietary and plasma cholesterol levels reduced α- and β-secretase activity and the amount of all APP-derived fragments in an APP gene-targeted mouse model [49]. The authors also suggest that the clearing mechanism, including ApoE, may be more active in elevated cholesterol levels. Thus, the specific lipid and protein background may influence outcome at many levels, indicating considerable complexity of AD pathology.

### The effect of cholesterol on APP

**Fig. 5:**
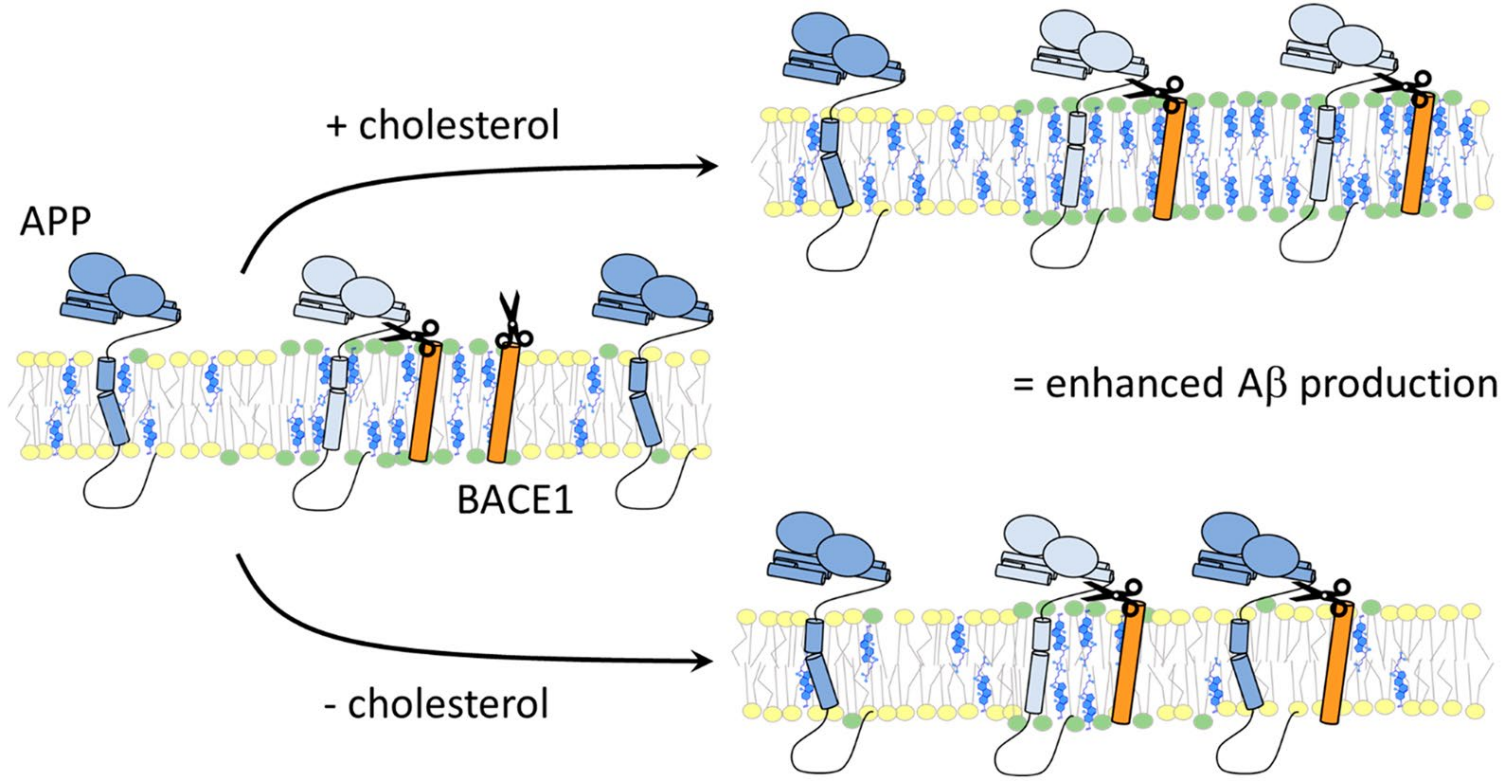
Effect of cholesterol content on APP processing and Aβ generation. APP is processed by β-secretase (BACE1) predominantly in cholesterol-rich lipid rafts. However, most of APP is localized outside rafts, and only a small fraction of APP is processed via the amyloidogenic pathway. An increase in cholesterol leads to increased raft organization, increased APP-BACE1 association within raft domains, and Aβ production (+ cholesterol). Another model suggests that lowering cholesterol levels may also promote amyloidogenesis (− cholesterol). Low cholesterol level leads to a lower number of rafts and lower amount of BACE1 in the rafts. As a result, BACE1 leaves the raft fraction and encounters its non-raft substrate, APP. Green circles, raft domain; yellow circles, non-raft area

In contrast to secretases, APP shows a more dynamic cellular localization. APP has been found in the PM, endocytic compartment, ER and GA. The specific distribution of APP in subcompartments such as mitochondria-associated ER, trans-GA or lipid rafts is closely related to the differential processing of APP [11, 221, 244, 245, 278, 299, 306–308]. For amyloidogenic cleavage, APP must colocalize with β- and γ-secretases in the i.c. cholesterol-dependent lipid rafts, whereas outside the rafts APP favors α-secretase-mediated processing [69, 227, 288, 293, 306, 308–311]. The importance of astrocytic cholesterol bound to ApoE for neuronal Aβ production was highlighted in a study by Wang et al. [81]. When cholesterol synthesis was inhibited in astrocytes, the size and number of lipid rafts in the neuronal PM decreased. And, although the association of β- and γ-secretases with rafts remained unchanged, APP-raft colocalization and Aβ production decreased. Cholesterol deficiency negatively affects endocytosis and BACE1-dependent APP cleavage [297, 311]. Because cholesterol binds to APP and the action of secretases depends on cholesterol, Aβ formation is closely related to cholesterol distribution [202, 229, 258, 293, 312–316]. Some data seem contradictory in the context that APP is better processed by BACE1 after cholesterol depletion when the enzyme leaves the rafts and binds to its substrate, the non-raft APP [169, 317] (Fig. 5). Cholesterol also affects APP dimerization and thus Aβ production [318], as described later. Different experimental conditions and cholesterol manipulations may lead to different results because cholesterol affects not only the distribution, stability, and activity of secretases and APP-C99 but also other raft-dependent processes in the cell.

Lowering cholesterol levels usually results in decreased APP-raft association and reduced Aβ formation [81, 243, 288, 294, 319]. However, more subtle alterations in cellular cholesterol levels must also be considered. Redistribution from the cytofacial to the extracellular/intraluminal face of the membrane was associated with decreased γ-secretase-mediated processing of C99 and Aβ production [320]. Furthermore, not only the direction, but also the extent of cholesterol change may affect Aβ production or Aβ degradation. A reduction of cholesterol level of more than 35% was associated with decreased Aβ secretion in CHO cells. However, under less than 25% reduction, Aβ secretion and AD pathology increased that was observed also in hippocampal membranes of rodents or AD patients where disturbation of lipid rafts led to increased APP-BACE1 colocalization outside the rafts and decreased raft-dependent Aβ degradation [169, 317]. It is important to mention here that model systems with increased APP content may lead to biased results because overloaded APP may artificially partition in rafts enriched in β- and γ-secretase [317, 321].

### APP binding to cholesterol

**Fig. 6:**
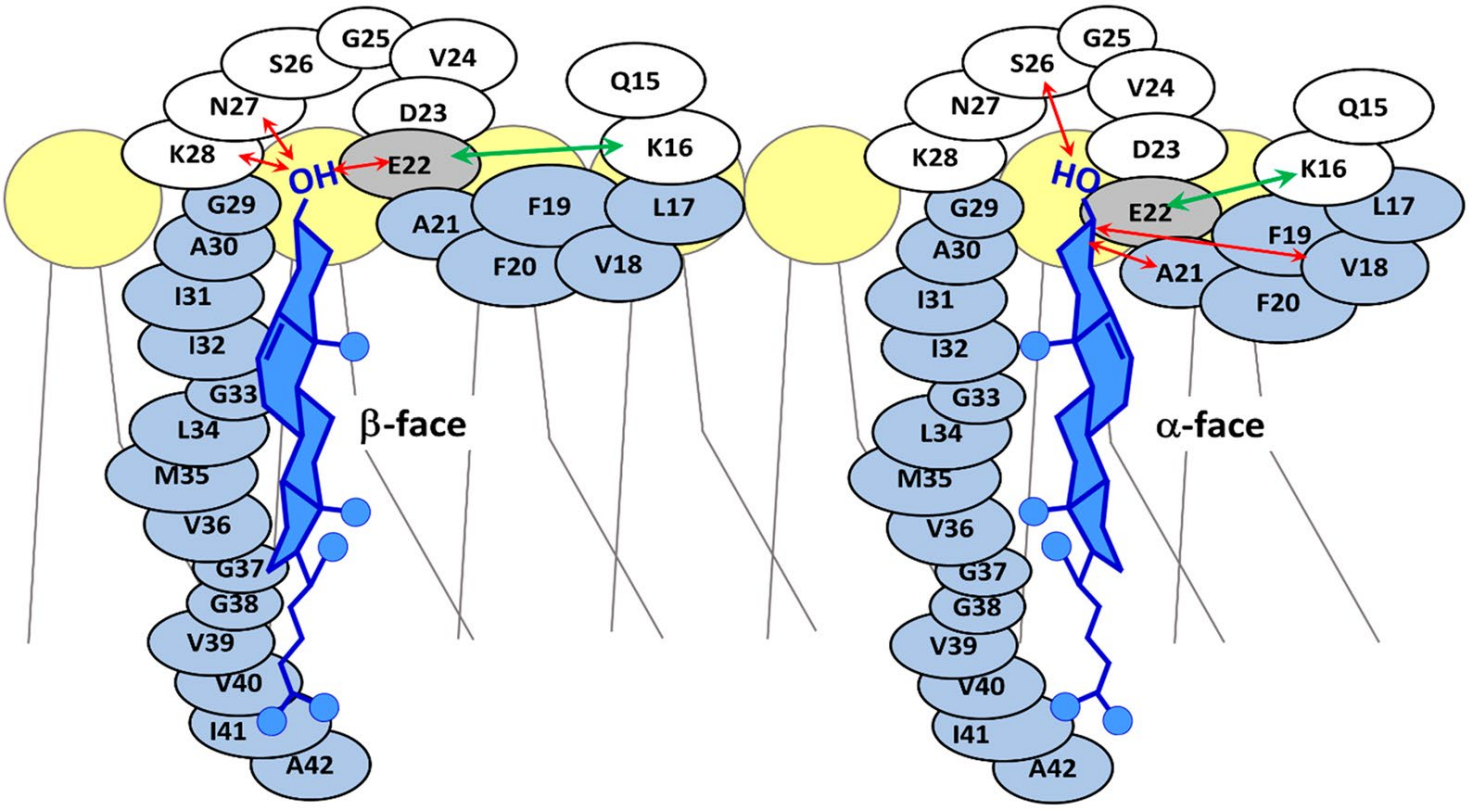
Interaction of C99 with cholesterol at the luminal/extracellular membrane face. In the j.m. region, both H-bonds and hydrophobic interactions between the cholesterol hydroxyl or aromatic A-ring and surrounding amino acids may be involved, as is indicated by red arrows. The exact arrangement depends on many factors, including the lipid environment, bilayer thickness, pH, and the tertiary and quaternary structure of the peptide. Adjusted according to [202, 310, 315]

The interaction of APP-C99 with cholesterol may support the localization of C99 in lipid rafts where amyloidogenic processing occurs [322]. Computational modeling revealed up to six binding sites for both the α- and β-faces of cholesterol in the APP-C99 structure, which differ in their affinity and interaction dynamics [322]. A simulation model showed two possibilities of C99-cholesterol interaction. In the tight conformation, the smooth α-surface of cholesterol binds tightly to Gly-x-x-x-Gly motifs through van der Waals interactions, whereas in the loose arrangement the rougher β-site faces C99 [202]. The mutual position of cholesterol and APP is stabilized by the H-bond between the cholesterol –OH group and the e.c. oriented amino acids, including Asn27 and Glu22 (Fig. 6). This interaction provides a pH sensor showing the pH dependence of Aβ production [310, 313]. While charged Glu does not reach the bilayer at neutral pH, accepting a proton in acidic environments renders it neutral and incorporates it into the bilayer, where Glu22 interacts with cholesterol and positions it in a specific orientation [310]. In the model of Nierzwicki and Czub, the H-bonding of Glu22 with cholesterol is not observed, but the interaction between Lys16 and Glu22 keeps the j.m. region within the bilayer and in close proximity to the membrane cholesterol, allowing mutual interaction [202].

By binding to APP, cholesterol promotes the amyloidogenic pathway by impeding the targeting of the cleavage site to α-secretase [313]. Mutation of Lys28 (K28A) disrupts the APP-cholesterol interaction and allows γ-secretase to continue cleavage and produce shorter forms of Aβ instead of the more dangerous longer Aβ40-42 [289]. Besides Lys28, also j.m. Lys16 strengthens cholesterol interaction with APP-C99. Moreover, Lys53 and Lys55 play similar roles at the cytofacial leaflet [315]. The authors conclude that no stable APP-cholesterol dimers persist over time, but various short-lived complexes with low interaction energy exist in the membrane. The exact form and lifetime of such heterodimers depends strongly on pH, membrane thickness (corresponding to the APP-C99-bilayer tilt), the presence of other lipids, and especially on cholesterol concentration or raft localization [315].

### Cholesterol and APP-C99 dimerization

A cholesterol content of more than 15% in the lipid raft pool is sufficient to disrupt C99 dimerization and trigger the monomer-dependent γ-processing [200]. This result is also supported by computational modeling [316, 318]*.* Electron paramagnetic resonance and Förster resonance energy transfer methods showed that C99 monomers bind to cholesterol at physiological concentrations and promote association with rafts and amyloidogenic processing of C99. However, when C99 membrane concentration increases and cholesterol levels are low (e.g. in the endosomal or GA fraction), C99 dimerization may occur, suggesting competition between C99–C99 and C99–cholesterol binding [250]. In molecular dynamics simulations, a low cholesterol content (10%) resulted in a stiffening of the bilayer and stabilization of C99 dimerization. At higher cholesterol levels corresponding to lipid rafts (40%), and especially in the outer membrane leaflet, cholesterol molecules became wedged in the upper part of the dimer and disrupted C99-C99 interactions, leading to C99 monomerization. This was linked to the exposure of the Gly-rich TM motifs to the surrounding cholesterol molecules [318]. From this study, it can be concluded that low cholesterol concentration stabilizes C99 dimers in the rigid membrane, whereas higher cholesterol concentration destabilizes them. In human iPSC-derived neurons and transfected HEK cells, cholesterol competed with APP dimerization, and statin-induced cholesterol reduction enhanced APP dimerization and decreased Aβ production, likely due to obscuring APP amino acids essential for β-secretase action at APP [323]. A complex relationship between cholesterol content, cellular trafficking, APP-BACE–γ-secretase interaction, and endosome-dependent Aβ production was proposed in a model by Feringa and Kant. High ER cholesterol prevents APP dimerization and causes transport of the monomeric APP into the PM. There, cholesterol stimulates raft clustering of APP, β- and γ-secretase, and clathrin-mediated endocytosis leading to amyloidogenic processing of APP to Aβ [324]. At the same time, dimerization of APP is associated with its retention in the ER and reduced maturation and processing of APP [323].

### Cholesterol esterification and APP processing

In addition to cholesterol itself, cholesterol esters (CE) formed in the ER might also play a role in amyloidogenesis, because CE levels are correlated with secreted Aβ levels [37]. It has been reported that reducing the amount of CE suppresses AP processing and Aβ production [323, 325, 326]. The reduction in cholesterol esterification results in more cholesterol molecules remaining in the ER. Because the ER cholesterol population represents the reference cholesterol pool, the cell responds by inhibiting cholesterol synthesis and uptake. This leads to reduction of cellular cholesterol amount along with impairment of cholesterol-dependent processes, including β- and γ-secretase activity toward APP-C99 [327]. Decreased synthesis of CE and increased cholesterol content in the ER increases retention of APP in the ER and reduces exposure of APP to β-/γ-secretases and Aβ formation [328]. Although these results are in contrast to the model of Ferringa and Kant (2021), they again illustrate the complexity of cellular processes that depend on cholesterol amount and distribution.

### Regulation of cholesterol homeostasis by APP

Importantly, APP itself can serve as a cholesterol-sensitive and regulatory element that reverses the relationship between APP and cholesterol content [329]. In astrocytes responsible for brain cholesterol homeostasis, deletion of the APP gene impaired lipoprotein and Aβ endocytosis, decreased i.c. cholesterol, and activated genes connected with cholesterol synthesis and uptake [330]. APP may affect the function of lipoprotein receptors and vice versa, as both proteins form a functional complex in the PM, secretory, and endosomal compartments [331–333]. Increased cholesterol content in the PM supports APP monomerization and internalization, resulting in enhanced i.c. APP processing and AICD release. Because AICD affects the transcription of genes connected with cholesterol metabolism, APP serves in this way as a cholesterol sensor in the cell [200, 201]. In the ER, by binding cholesterol, the C99 pool may contribute to the formation of ER-lipid rafts involved in cholesterol esterification, a process closely associated with cellular sterol homeostasis [334].

## Closing remarks

In the last decade, many findings pointed to the importance of a cholesterol-rich environment for β- and γ-secretase-mediated processing of the amyloid precursor protein, APP. All steps of Aβ production, including stability and activity of secretases, their aggregation with substrate, and association with the endosomal compartment and lipid rafts, have been found to be determined by cholesterol levels. However, because so many studies have focused on the role of cholesterol in amyloidogenesis, a variety of inconsistent results have been obtained. This fact suggests that cholesterol plays multiple roles in membrane-associated processes. Many proteins involved in Aβ formation contain different cholesterol-binding sites, so that the binding of cholesterol affects not only their conformation but also their targeting to other members of the machinery and distribution to specific compartments in which other partners are differentially represented.

Nonamyloidogenic α-secretase processing prevails in non-raft plasma membranes, where a significant pool of APP is also located. The opposite is true for β- and γ-secretases, which are concentrated in i.c. compartments (especially endosomes) and in cholesterol-rich lipid rafts. In this context, cholesterol has two essential roles: (1) by direct binding, it affects protein conformation and function, and (2) it establishes specific raft environment. Therefore, any change in cholesterol content may directly or indirectly influence membrane processes, and furthermore, these effects can be contradictory and depend on cholesterol concentration and the presence of other lipids and proteins. The use of different experimental and theoretical models not only leads to controversial results, but also allows us to understand an intricate web of relationships between all the players involved in APP processing. This is well illustrated by the effects of cholesterol alteration on increased APP-β-secretase colocalization after both cholesterol lowering and cholesterol elevation (Fig. 5), underscoring the fact that any disruption of cholesterol homeostasis may exacerbate the onset and progression of AD.

It has been established that APP can exist in monomeric or multiple dimeric forms, which have a strong influence on membrane localization and interaction with secretases. The relationship between the distinct APP and C99 forms, cholesterol, and cholesterol-dependent processing appears to be very complex, as different and conflicting results have been obtained. Both APP-C99 monomers and dimers are suitable substrates for secretases; however, processing may differ in the length of the final amyloid β product, reflecting the different spatial orientation of the substrate and γ-secretase. The dependence of APP cleavage on localization in the plasma membrane or intracellular environment after cholesterol-dependent endocytosis brings up another point where cholesterol is involved in Aβ formation. The involvement of the ER and GA in the amyloid-producing machinery further complicates the understanding of the regulation of amyloidogenesis. Certain forms of APP and C99 are differentially recognized as substrates for inter-organelle transport. The influence of cholesterol on APP-C99 dimerization therefore underscores the complexity of the system, which contains various monomeric or dimeric APP and C99 forms that may be bound to cholesterol and localized to the raft or non-raft membranes of different organelles where different secretases are present. Cholesterol binding to the APP-C99 monomer may attract the peptide to the β-/γ-secretase-rich environment of lipid rafts, whereas the dimeric form may remain longer in the vicinity of β-/γ-secretases in endosomes because the dimer is not recognized by SORL1 for recycling to the TGN. The other source of complexity is due to the specific APP-C99 position and orientation in the membrane, which results in differential exposure of cleavable sites for β-/γ-secretases. Cholesterol can obscure these sites, which are then more accessible in the dimeric state, or it can expose them by disrupting the closed conformation of the dimers. Thus, different interactions of varying importance between cholesterol and APP-C99 have been found, reflecting the application of different models and introducing a great deal of uncertainty into our view of the arrangement. However, the presence of different arrangements provides an explanation for the high sensitivity of APP-C99 processing to more or less significant alterations in the lipid environment, because only minor changes can cause significant conformational shifts and resulting secretase actions.

In summary, cholesterol influences amyloidogenesis at several levels, depending mainly on the nature of its interaction with protein partners. Because a specific lipid environment plays a crucial role in this complex process, all computational models and artificial membrane systems, although very useful in revealing molecular details, provide incomplete data when compared with real neuronal membranes. Unfortunately, no samples of the early stages of AD from affected human brain parts are available for functional analysis of Aβ production. Besides cholesterol, which affects the fluidity and thickness of the bilayer, other lipids or fatty acids may also affect Aβ formation. In particular, PUFA, which reduce membrane rigidity and have heterogeneous molecular shapes, have effects on the protein machinery involved in amyloid production. Monounsaturated oleic acid and ω3 docosahexaenoic acid have been shown to be protective, whereas ω6 PUFA or saturated FA exacerbate the AD pathology [335, 336]. PUFA also enhanced cholesterol-SM association by excluding more cholesterol molecules from bulk membranes into the raft fraction [101, 105, 337].

Consideration of the complexity of the interactions is critical because all simplified models, by their nature, represent only a partial aspect of the problem. However, each partial result helps to untangle the complex web of relationships between molecules responsible for one of the most devastating diseases of our time. In the future, the use of neural stem cells and molecular genetic techniques to manipulate cholesterol levels and other lipids and proteins involved in the APP processing system may help us unravel the intricate web of relationships between Aβ formation and cholesterol distribution.

## Data Availability

Not applicable.
